# Using economic value signals from primate prefrontal cortex in neuro-engineering applications

**DOI:** 10.1088/1741-2552/ae0bf6

**Published:** 2025-10-15

**Authors:** Tevin C Rouse, Shira M Lupkin, Vincent B McGinty

**Affiliations:** 1 Center for Molecular and Behavioral Neuroscience, Rutgers University-Newark, Newark, NJ, United States of America; 2 Department of Neurobiology, University of Chicago, Chicago, IL, United States of America

**Keywords:** brain–machine interface, decision-making, deep learning

## Abstract

*Objective.* Brain–machine interface (BMI) research has shown the efficacy of using motor and sensory-related neural signals to assist physically impaired patients. Despite the comparable ability to extract more abstract cognitive signals from the brain, little effort has been devoted to leveraging these signals in neuro-engineering applications. In this study, we explore the use of neural signals related to economic value, a key cognitive construct, in a BMI context. *Approach.* Using multivariate time series data collected from the orbitofrontal cortex in non-human primates, we develop deep learning-based neural decoders to predict the monkeys’ choices in a value-based decision-making task. We implement a reinforcement learning-based training approach to develop adaptive decoders that can be extended to handle multi-step decisions, which frequently arise in real-world settings. *Main results.* We develop neural decoders leveraging subjective value signals to predict the monkeys’ choices with ${\gt}70\%$ accuracy on average, with above-chance accuracy even when choice options are objectively equal. We show that this same decoder architecture can be trained to execute choice-related actions and execute action sequences aligned with the user’s goal. Finally, we explore a decoder architecture that uses a neural forecasting model equipped with task-related information, and show that it makes high accuracy predictions ${\sim}300$ ms sooner than would otherwise be possible. *Significance.* These findings support the feasibility of user preference-informed neuro-engineering devices that leverage abstract cognitive signals to aid users in goal-directed behavior. They suggest that using abstract cognitive signals in real-world settings may be more accurate when combined with information from multiple sources, such as motor and sensory regions. This research also highlights the potential need for systems to measure their confidence in their actions when user input is minimal.

## 1. Introduction

Decoding neural signals is a primary focus of neuro-engineering applications. (B)y extracting the neural correlates of a particular cognitive process, neuro-engineering systems like brain machine interfaces (BMIs) can help restore or augment that process. For example, BMI systems can partially restore motor functions by decoding movement-related signals from cortical motor areas, allowing users to control robotic limbs [1] or on-screen cursors [2]. Recent work has expanded on these ideas to decode neural correlates of sensory signals [3]. Other work in non-human primates has focused on invasive BMI applications for the prefrontal cortex to decode signals related to eye movements and spatial locations in a virtual maze [4]. Despite these advances, very few studies have explored the use of abstract cognitive signals related to subjective mental contents [5]. By detecting signals that are related to a user’s goals or beliefs, systems can be developed to aid higher cognitive functions.

In this work, we seek to explore the development of BMI systems that can extract abstract neural signals encoding economic values. Economic value (utility) is the subjective perception of the benefit or harm associated with a given stimulus, outcome, or course of action. Values are typically learned through experience and depend on an individual’s behavioral context, goals, and internal state. Neural representations of value form the foundation for goal-directed decision-making [6]. Thus, the ability to extract subjective value information from neural activity is an essential step in decoding abstract mental contents such as goals and desires. The neural correlates of value have been extensively characterized in animal models, including non-human primates [7]. Thus, this work seeks to operationalize neural representations of subjective value to explore the development of value-based BMI systems.

Much of the work on the neural representation of subjective value has focused on the orbitofrontal cortex (OFC). This prefrontal cortical region is critical for value-driven behaviors. Not only do OFC neurons encode economic value, but disrupting this encoding disrupts many kinds of decision-making behavior, in both humans and animal models [8]. Given the role of OFC in the decision-making process, our objective is to assess whether OFC-derived signals can be used in a BMI context to help the user achieve their goal. Previous studies [9, 10] have demonstrated that neural decoders with access to neural activity from groups of OFC cells can be trained to predict the value of items as well as which items the animal will choose.

Although these studies provide insight into the neural mechanisms of value encoding, they have not explored the use of such signals in a BMI context. With this as our motivation, we will deviate from these studies by proposing an alternative approach to decoding intended choices at fast latencies from the activity of OFC neural ensembles. We take a methodological deviation from previous value-decoding studies [9, 10] by using a reinforcement learning approach [11] to develop value-based neural decoders. This approach allows us to: (1) account for the context-dependent and dynamic nature of value signals, (2) update the decoder with changes in a participant’s subjective evaluations, and (3) develop decoders that can make predictions in multi-step processes. These abilities are likely to be critical in real-world settings where a value-based BMI would be used.

To thoroughly assess value decoding in a BMI context, we explore three hypothetical scenarios using an existing data set from monkeys performing a simple economic decision task. Scenario 1 involves learning to predict the monkey’s subjective decision with access to the neural activity within a trial. This scenario provides insight into the basic capabilities of a value-based neural decoder in handling single-action processes. Scenario 2 will require the neural decoder to learn to execute action sequences that align with the monkey’s choice preferences. This scenario gauges whether value-based neural decoders can learn to assess how their actions in multi-step scenarios can benefit the user, despite their actions not being explicitly indicated by the user. Scenario 3 will require the agent to make choice predictions early in a decision trial, when fully informative neural signals are not yet available. This scenario allows us to assess the limits of the decoder’s basic predictive capabilities and explore how it can be augmented to improve its predictive performance when it has incomplete information. Within these three scenarios, we will also explore four factors important for value-focused BMI systems: the influence of trial count on predictive performance, the identification of what value information is preserved when the focus is on choice prediction, the sources of uncertainty that influence our decoder’s performance, and the potential tradeoffs that come with different training approaches (i.e. supervised learning vs reinforcement learning).

We observed that we could obtain above-chance predictions on which item the monkey desired, including in cases where neither item was objectively better. Furthermore, we also achieved above-chance performance in the multi-step case where the agent had to learn action sequences aligned to the monkey’s choice preferences. We also observed that using forecasted neural activity allowed the decoder to make choice predictions sooner, but only when other information about the task conditions (i.e. the stimuli present in each trial) was incorporated into the forecasting process. When we compared our RL approach to a standard supervised learning approach, we observed that the RL construction provided improved accuracy in early-stage choice predictions.

## 2. Methods

### 2.1. Problem description

This study aims to design a value-based BMI system that learns to predict and incorporate the user’s choice preferences in its action selection. We tested this design by building a neural decoder that uses the neural activity collected from monkeys performing a two-alternative economic decision task (a subset of the data used in [10]). On each trial, the decoder takes a multivariate time series (i.e. spike count data from a group of cells over time) and extracts the relevant value signals needed to predict the monkey’s choice. Note that the monkey’s choice may or may not be the best option available on the trial.

**Figure 1.**
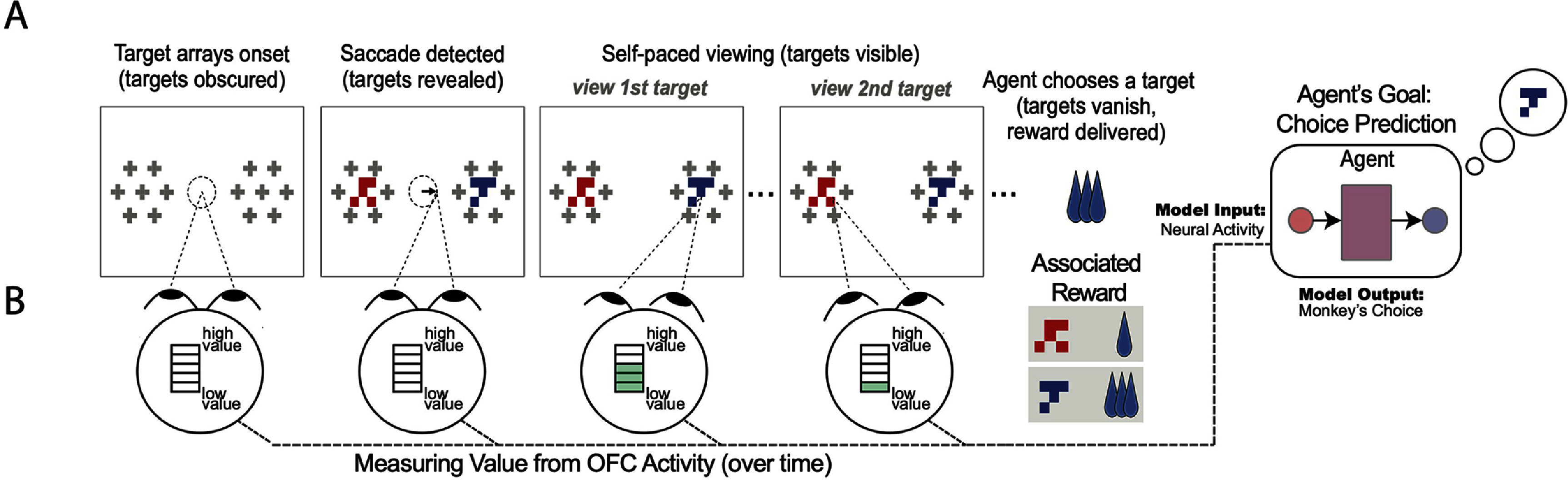
Economic value-based BMI (brain machine interface) framework. (A) Shown is a two-alternative forced-choice economic decision task that the monkeys performed while OFC neural activity data was collected. For task and behavior details see [12]. The monkey initiates a trial by fixating on a dot in the center of the screen, after which two covered stimuli appear. When the monkey saccades towards one of the stimuli, the stimulus in the direction of the saccade is revealed. Crowders (i.e. the visual elements surrounding each stimulus) force the monkey to look at the stimulus to gain information from it; they cannot identify the stimuli using peripheral vision. Each stimulus is associated with a reward ranging from 1 to 5 drops of juice; physically distinct stimuli can have the same reward association, meaning that in some trials there is no objectively better (largest reward) choice. Monkeys press a lever to indicate their choice, after which they receive the associated juice reward. (B) We consider a hypothetical scenario where a neural decoder extracts information from the neural activity obtained from the monkey while it is examining the stimuli. The decoder is tasked with extracting value information related to the stimuli and predicting the monkey’s choice. See figures 2(A) and 5(A) for model architectures tested in this study.

**Figure 2.**
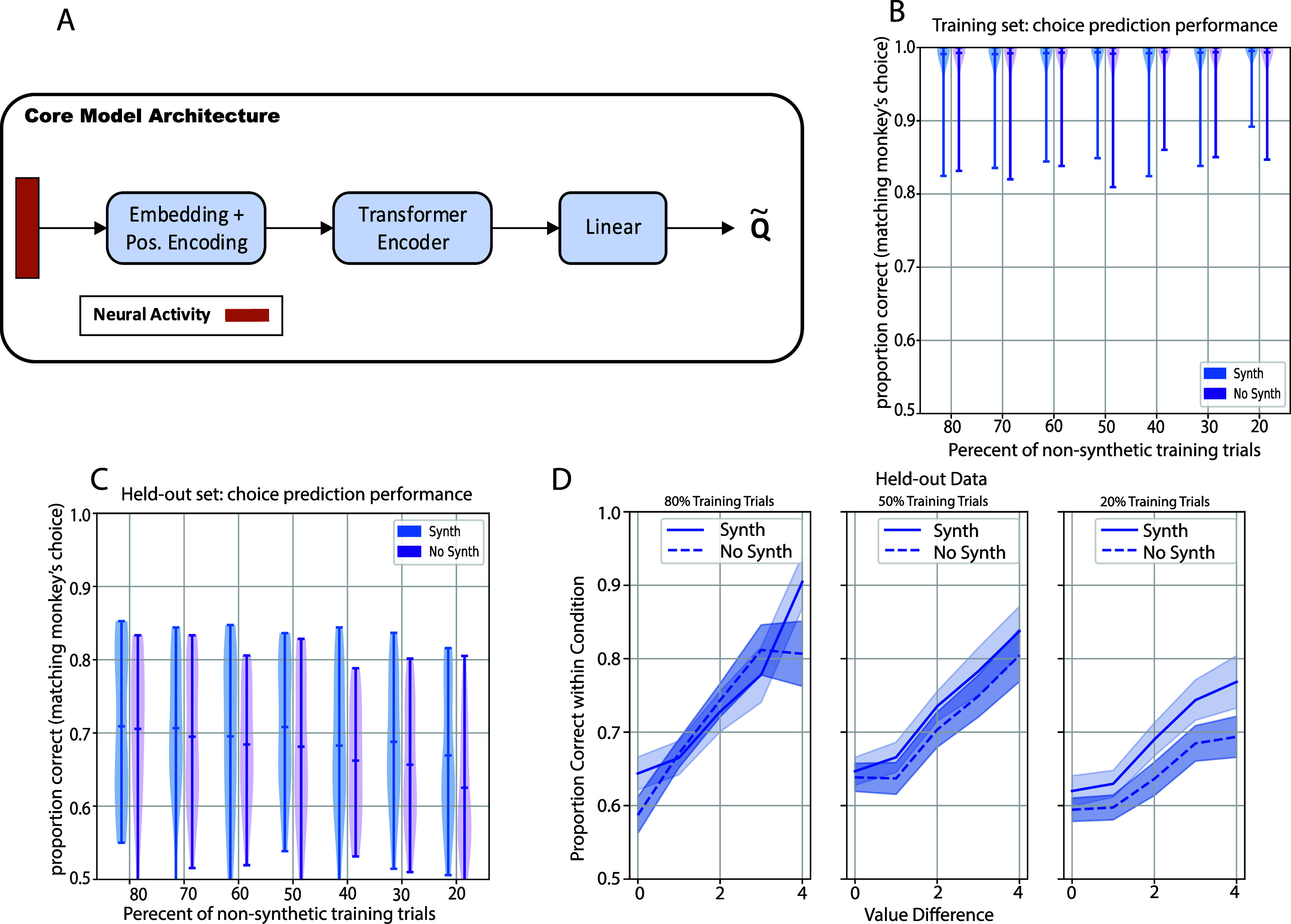
The reinforcement learning agent can learn to predict the monkeys’ choices. (A) Shown is the Core model architecture used for the agent. The agent model is designed to take as input neural activity (spike counts over time) and predict the *Q*-values associated with each state-action pair. (B) Distribution of the agent’s choice prediction performance on the training data at the end of training. Performance is reported on the case when the agent is trained with and without synthetic data (see Reinforcement learning approach section (2.4) in the methods). The central bar in each violin plot corresponds to the mean performance over sessions, while the bars on the edges correspond to the range. The *x*-axis gives the percentage of trials used in the training data for each session. There were no significant differences between Synth and NoSynth training approaches (*p*-values were ${\gt}0.05$ for the 80, 70, 60, 50, 40, 30, and 20% cases using a paired *t*-test (*df* = 22) comparing Synth vs NoSynth; table 1). (C) Distribution of the agent’s performance on the held-out data over sessions. Plotting conventions are the same as in (B). Performance was higher when synthetic data was used in runs using 50%, 30%, and 20% training data (*p*-values were ${\gt}0.05$ for the 80, 70, 60, and 40% cases, *p*-values were $ < 0.01$ for the 50, 30, and 20% cases using a paired *t*-test (*df* = 22) comparing Synth vs NoSynth; table 2). (D) Performance of the agent on the held-out data as a function of the value difference condition for the 80%, 50%, and 20% training data cases with and without synthetic training data. The shaded region corresponds to session-wise SEM.

We considered two versions of this hypothetical scenario. The first version is when the decoder has access to the complete multivariate time series (i.e. all time bins; figures 1 and 2, see Environment 1 in methods section 2.4, Reinforcement learning approach). As stated above, the decoder must learn to predict the monkey’s choice. In the second version, the decoder is provided a continuous segment of the multivariate time series up to time point *t* < *T* (figures 5 and 6; see Environment 3 in methods section 2.4, Reinforcement learning approach). For this version only, we test the performance of two different model architectures, Core and Internal model, described below.

**Figure 3.**
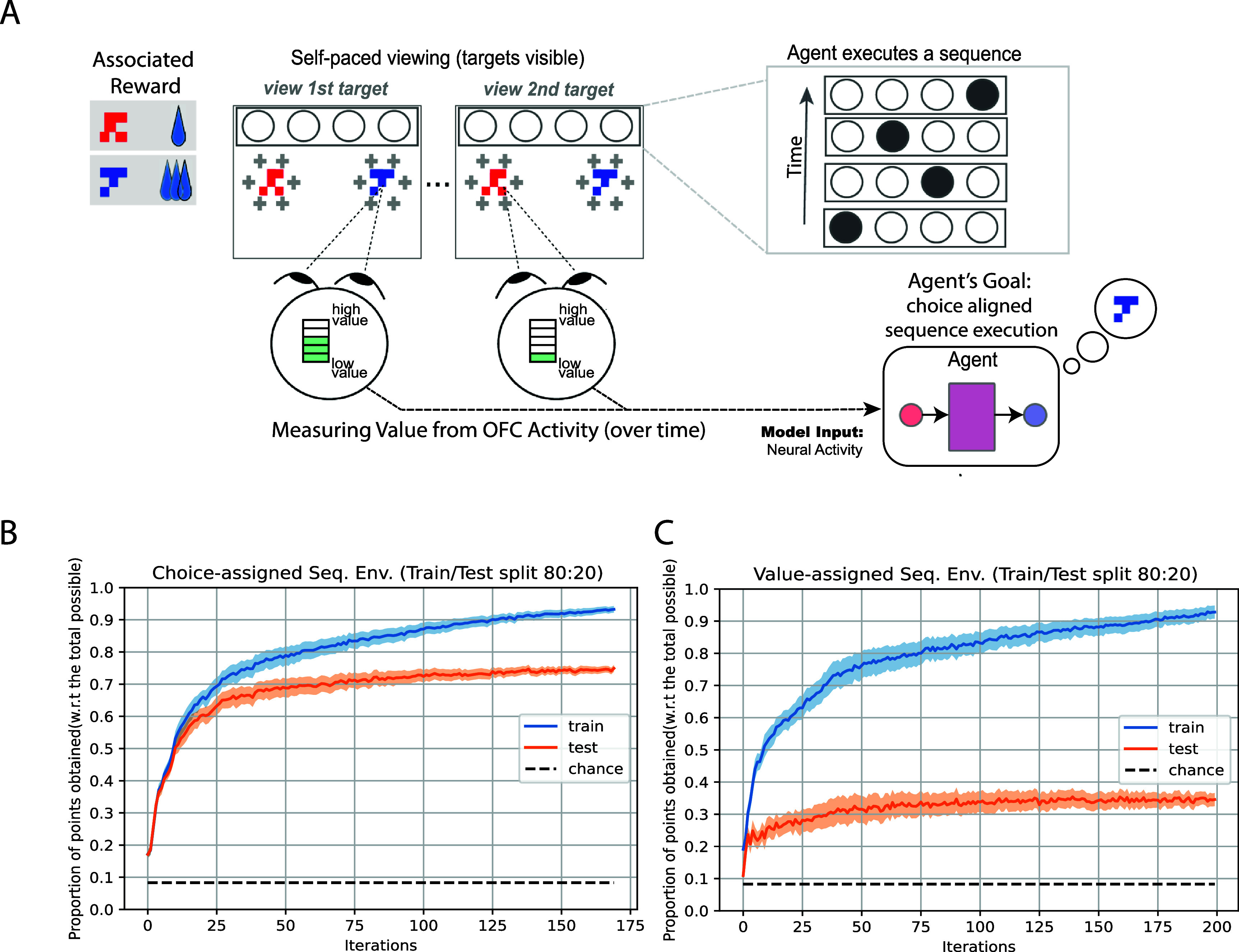
The agent learns to extract information about the monkeys’ choices to make goal-aligned decisions (A) Shown is a variation of the two-alternative forced choice economic decision-making task described in figure 1. In this hypothetical task, the monkey receives a reward if the monkey selects the appropriate lever and the agent selects the correct action sequence (illustrated by the circles at the top). See methods Reinforcement learning approach (2.4), Environment 2 for details. The agent must learn to simultaneously decode the monkey’s choice and execute the appropriate target sequence. We consider the case where the sequence depends on the target’s value level (i.e. five unique sequences) or the choice (i.e. two unique sequences). (B) The proportion of the reward achieved by the agent (normalized by the total possible within the environment) in the choice-assigned sequence case. We only consider the case where the model was trained using 80$\%$ of the training data. Chance was computed using the average performance of a random agent interacting with the environment, averaged over 100 episodes. (C) Like panel B, but examining performances for the value-assigned sequence case.

We also considered a variation of the task that explored how value-related choice signals could be used to guide learning of indirect, but goal-aligned actions. We considered a hypothetical task (figure 3; see Environment 2 in methods section 2.4, Reinforcement learning approach) that required the agent to learn action sequences aligned to the monkey’s choice. On a given trial, each target was assigned a sequence. We assigned a sequence to a target based on the target’s value level (e.g. 1, 2, 3, 4, or 5) or the order in which the monkey viewed the target (first or second). Within our hypothetical scenario, we considered the monkey having received the reward if the decoder executed the correct action sequence assigned to the target. Note that the idea of action sequences discussed in this hypothetical task was not part of the original experiment from which this data was collected, but an aspect added to the data post-data collection. See the Behavioral task section (2.2) in the methods for details on the original experiment.

### 2.2. Behavioral task

We use data from [10], which used the following two-alternative forced-choice task to understand the neural mechanisms of value-based decision making. In this task, monkeys were tasked with examining two items on a display and selecting one (figure 1(A)). Each item, upon being chosen, yielded a fixed amount of juice reward of 1, 2, 3, 4, or 5 drops. On each trial, the monkeys were allowed to examine each option as long as they wanted, and to choose when ready. When the reward associations of the two items differed by two drops or more, the monkeys almost always chose the larger item; but when they were equal or near-equal, the monkeys’ decisions were variable from trial to trial (see [12], their figure 1(D)).

### 2.3. Data and preprocessing

We use 23 sessions of neural data collected from two rhesus macaques performing the value-based decision-making task described above (Monkey C: 10 sessions, Monkey K: 13 sessions); this is a subset of the data reported in [10]. All data collection procedures were performed according to the NIH Guide for the Care and Use of Laboratory Animals and were approved by the Animal Care and Use Committees of Stanford University (protocol number 9720) and Rutgers University—Newark (protocol number McGinty$\_00861$). For this work, we only used cells that were identified to be in the posterior portion of the OFC. We excluded sessions with fewer than 10 cells and fewer than 400 trials. All analyses were performed within each session’s data. For a given session, the neural data consists of spike count data collected from single neurons in the OFC (mean of 27.6, SEM 2.7 cells per session). Spikes from single cells were counted in 50 ms, non-overlapping time bins. Given the structure of the task, we examined data time-locked to when the monkey viewed the second target in each trial, which is the time point at which the monkey has acquired all the information necessary to make a decision (i.e. the identities of both targets). We used spike count data from 200 ms before viewing the second target to 400 ms after viewing the second target. The neural data in each session had the following structure: Trials × (Number of Cells) × (12 Time Bins of 50 ms each). The behavioral data includes the choice that the monkey made for each trial, along with the identity of both stimuli.

Generalizability for the trained agent was assessed by a series of random train-test splits, in which the fraction of training trials was varied between 20$\%$ and 80$\%$ in increments of 10$\%$. For each session, we perform only one train-test split at each of the training fractions tested. We split this data randomly since we had no reason to maintain the original trial order of the session. Since data was collected after the monkey was trained on a stimulus set (see [12], for details), we would not expect within-session learning effects if we had maintained the original trial order. Before model fitting and testing, we preprocessed the neural data as follows: for each cell and time bin in the series, we mean-centered and scaled the data to unit variance, and then scaled the data again to the interval $[-1,1]$. The statistics used in the scaling process were derived for each cell using the portion of the data used in training.

### 2.4. Reinforcement learning approach

We have chosen to frame our problem as an offline RL problem. In a typical offline RL problem, we have access to a dataset *D* consisting of tuples $(s, s^{^{^{\prime}}}, r, a)$ where *s* is the current state, $s^{^{^{\prime}}}$ is the next state, *r* is the reward, and *a* is the action taken by some agent. Each tuple represents an event where an agent interacting within an environment observed the state *s*, made an action *a*, received a reward *r*, and then was presented the next state $s^{^{^{\prime}}}$ in the environment. Within the context of our decoding problem, we consider the neural decoder to be our agent. Each session can be thought of as an environment where states are the neural activity observed on a trial. When the decoder predicts the option that the monkey chose on a particular trial (i.e. the decoder’s action), the decoder receives a reward and then is presented with the next trial. The general algorithm for training our decoder is given below.

**Table:** 

**Algorithm 1.** RL training procedure.
$E,\Psi,g,N_{I},N_{E},K,B$ $\rhd$Environment,Agent,Discount Factor,Number of Iterations,Number of Episodes,Sample size,Storage Buffer
**for** $n = 1, \dots, N_{I}$ **do**
$B^{^{^{\prime}}} \gets Explore(\Psi,E,N_{E}) = \{\{(s,s^{^{^{\prime}}},r,a)\}\}_{N_{E}}$ $\rhd$Agent executes *N*_*E*_ episodes in *E*
$B \gets B \cup B^{^{^{\prime}}}$
$S \gets SAMPLE(B,K)$ $\rhd$Randomly sample *K* tuples
$A \gets UPDATE(\Psi,S)$ $\rhd$Update the parameters of Ψ
**end for**

Exploration for all agents was implemented using a Boltzmann policy described by the following expression:

\begin{align*} p\left(a|s\right) = \frac{\mathrm{e}^{Q\left(x,a\right)/\tau}}{\sum_{a^{^{^{\prime}}} \in A}\mathrm{e}^{Q\left(s,a^{^{^{\prime}}}\right)/ \tau}}\end{align*}

where $Q(s,a)$ is the state-action value estimated by the agent. We set the temperature *τ* = 1.

To explore how value signals can be incorporated into a neuro-engineering context, we consider three different environment types for training an agent on data within a session. Each environment represented a hypothetical neuro-engineering scenario. Below are the details for each environment type.

**Figure 4.**
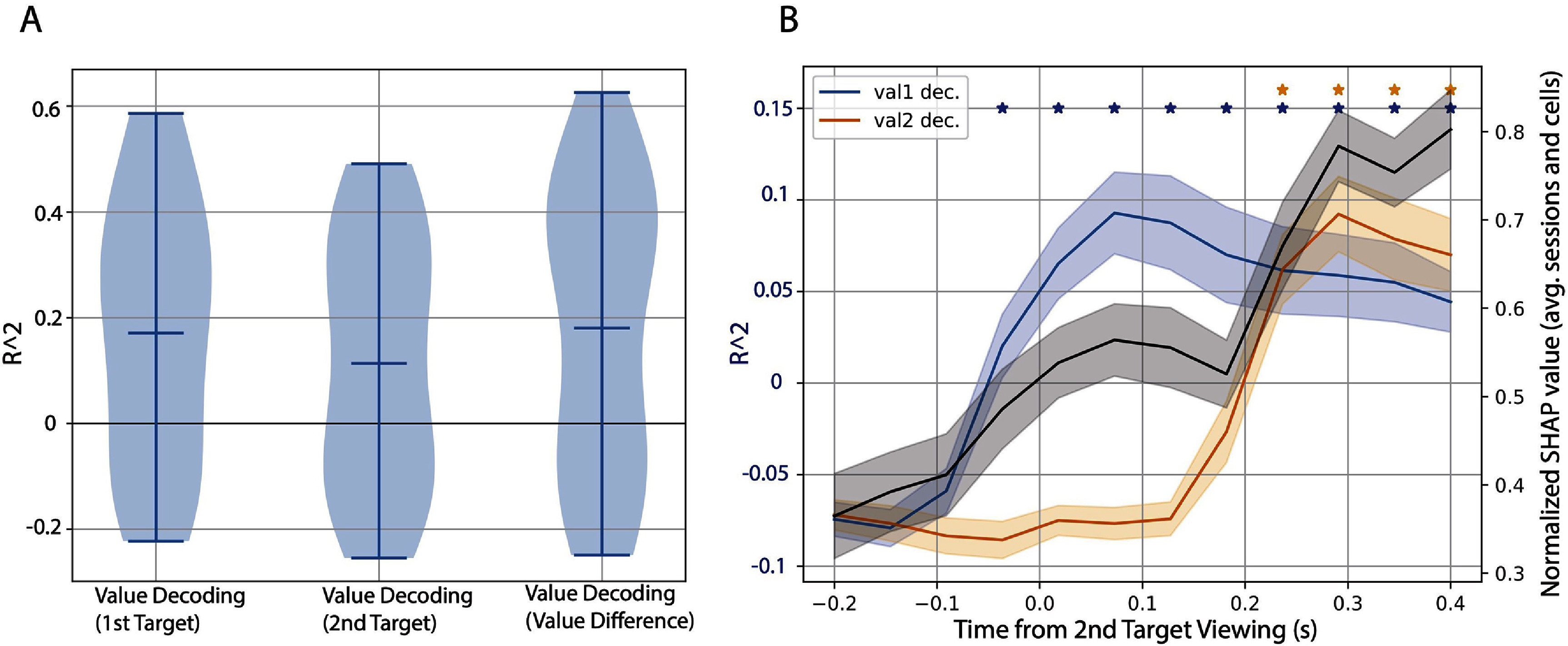
The agent predicts the monkeys’ choices using value information encoded near the times when each option’s value signal reaches a peak. (A) Decoding performance (*R*^2^) from the agent’s internal representation. A vector extracted from the transformer encoder’s output was used to decode the value of the 1st stimulus viewed, the 2nd stimulus viewed, and the value difference between the 1st and 2nd stimulus. Decoding of each value was done using OLS applied to the representations generated from 80% training data case. The distribution of *R*^2^ values is shown, with the central bar representing the mean and the edges corresponding to the minimum and maximum *R*^2^ value. Decoding performance of different value-related measures using the agent’s internal representation (comparing the *R*^2^ to 0 using a one sample T-test (*df* = 22): *p* < 0.05 for Value Decoding (1st Target), Value Decoding (2nd Target), and Value Decoding (value difference); table 3). (B) The blue and orange curves give the decoding accuracy for the value of the 1st-viewed stimulus (blue) and second-viewed stimulus (orange) using the original neural data over time. The black curve shows the Shapley values for the agent’s *Q* value predictions. The Shapley values were normalized within the session and averaged over cells and trials. Shaded areas correspond to session-wise SEM. Stars indicate statistical significance for decoding value (one sample *t*-test (*df* = 22) comparing the *R*^2^ to 0 at each time point: *p* < 0.001).

Environment 1 (used in figures 1, 2 and 4)

**Environment definition** Within this environment, the agent was required to predict which of the two presented targets the monkey chose.

**State definition** Each state corresponds to neural activity observed on a given trial. We added uniform noise (bound of 0.1 around 0) on each trial to create synthetic trial data to increase the size of the training set ([13]. This noise was added to the scaled data (see Data preprocessing section (2.3)) and clipped to maintain the $[-1, 1]$ value range. We explored values for the noise’s bounds between 0.1 and 0.2 but these variations were quantitatively similar.

**Reward definition** A reward of +1 when the agent correctly predicted the monkey’s choice and 0 otherwise.

**Training details** We trained the agent for 300 iterations. On each iteration the agent completes 5 episodes. We leveraged a *Q* learning approach where the target value for a particular state-action pair that the agent is trying to approximate is computed using the following expression:

\begin{align*} Q\left(s,a\right) = r + g * \mathrm{max}_{a^{^{^{\prime}}}}Q\left(s^{^{^{\prime}}},a^{^{^{\prime}}}\right).\end{align*}

The mean squared error (MSE) was used as the loss function. For this environment, we used a discount factor *g* = 0. The Adam optimizer was used for optimization [14]. We only used the Core model architecture for this environment. See Model architecture section (2.5) for more details.

Environment 2 (used in figure 3)

**Environment definition** This environment represents the hypothetical scenario in which the agent is required to identify the target the monkey chose and then execute an action sequence that is uniquely mapped to either the value of the chosen target or the order in which the target was viewed by the monkey. The action sequence was defined to be the selection of 1 of 4 possible actions chosen in a particular order. We fixed the length of the sequence to be 4. Note that the idea of action sequences used in this environment is not part of the original experiment from which this data derives, but is an aspect added to the data post-data collection. See the Behavioral task section (2.2) in the methods for details on the original experiment.

**State definition** We defined states to be the combination of neural activity (with uniform noise; see Environment 1 for details) observed on a trial and the current action (see figure 3(A)) encoded as a 1-hot vector. At the beginning of a trial, this vector is the zero vector. A new trial is given when either the agent executes the full sequence or when the agent makes a mistake on a sequence.

**Reward definition** The agent receives a +1 when it correctly chooses the appropriate action and 0 otherwise. On each trial the agent can receive a total of 4 reward points (+1 for choosing the correct action in the sequence). The maximum amount of points possible in an instance of this environment is equal to sequence length × number of trials.

**Training details** We used a *Q* learning approach to train the agent, like the training approach in Environment 1. The agent was trained for 170 iterations for the viewing order-mapped sequence case and 200 iterations for the value-mapped sequence case. We used a discount factor *g* = 0.99. We also maintained a target network, which was updated using the soft update approach [15]. To speed up the learning, we leveraged a state representation learning approach. We separated the agent’s architecture into 2 components: (1) a state representation component and (2) a *Q* function component. The state representation component served to reduce the neural data’s dimension [16]. The *Q* function predictor received as input the action sequence state vector as well as the choice-related representation learned by the state representation component. This allowed us to structure the representations for the behaving agent in a more efficient way compared to the end-to-end training approach that we used for Environment 1-trained agent. Both components were updated independently but trained simultaneously (i.e. a different optimizer per component). The state representation component was trained (in a supervised manner [17]) to predict the monkey’s choice using a cross-entropy learning loss function. The *Q* function predictor was trained using the RL approach described above with MSE as the loss function. For the value-mapped sequences case (figure 3(C)), we also incorporated a regularization term [18] into the loss function. Optimization was performed using the Adam optimizer. We only considered the Core model architecture for this environment. See Core model architecture in the Model architecture section (2.5) for more details.

**Figure 5.**
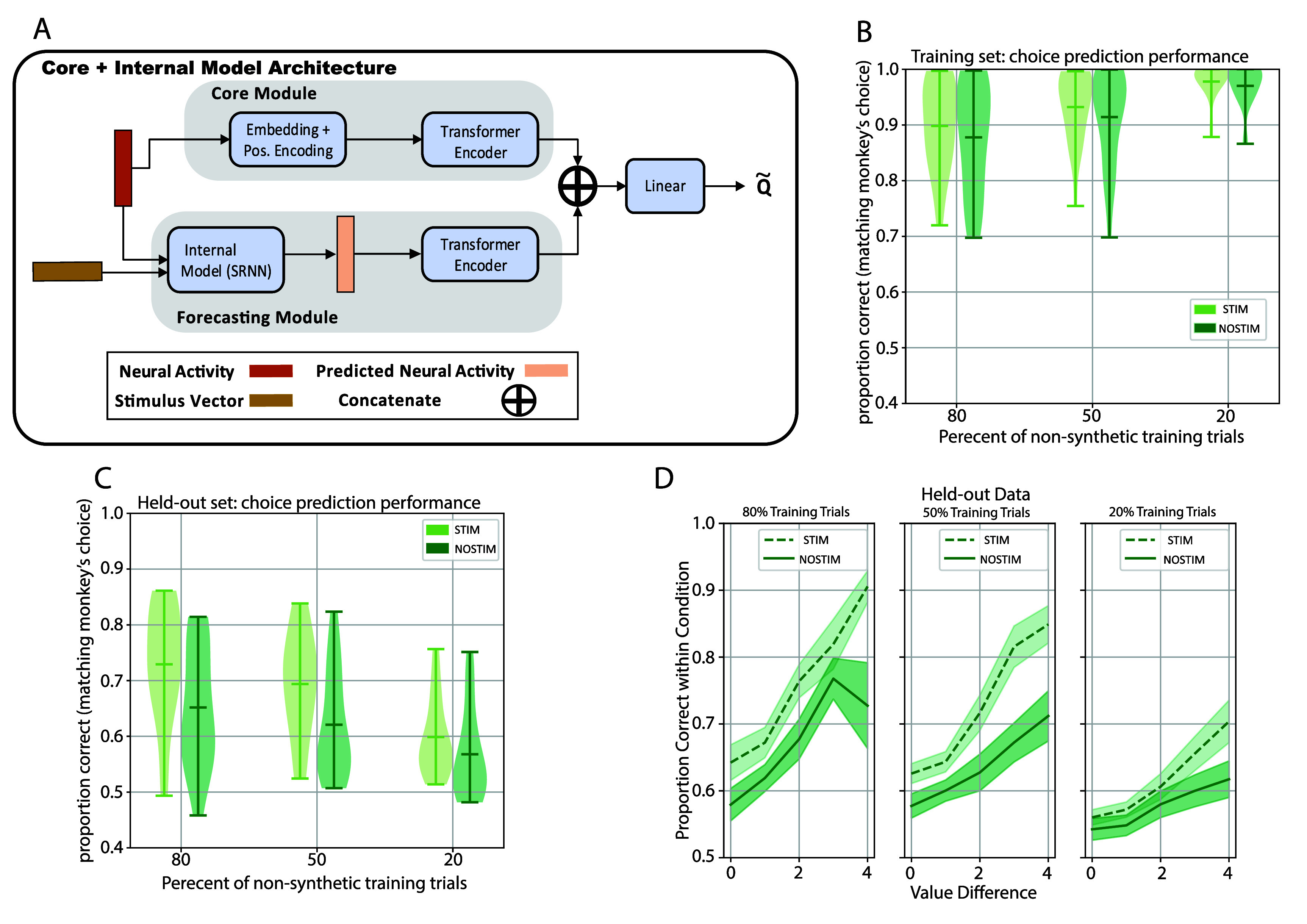
A reinforcement learning agent equipped with an internal forecasting model can learn to predict the monkeys’ choices. (A) The agent architecture consists of the same modules as the Core model (figure 2(A)) but also contains a processing structure for the Internal forecasting model. The Internal model receives both the OFC neural activity up to a given time point *t* and vectors indicating the stimuli presented to the monkey; together, these are used to forecast the neural activity that would occur in OFC after time *t*. Using the Internal model’s forecasts, the agent then predicts the *Q*-value for both state-action pairs. For clarity, we will refer to the modules found in the Core model as the Core module. The combination of the Internal model and the transformer encoder that uses the Internal model’s output will be called the forecasting module. (B) Distribution of the agent’s performance on the training data over sessions for the STIM and NOSTIM cases. The number of trials used in the non-synthetic training data for each session was determined by a percentage of the total trials. The central bar corresponds to the mean performance over sessions. The bars on the edges of the violin plots correspond to the minimum and maximum performance over sessions. Performance was significantly higher in the STIM condition (*p*-values were $< 0.005$ for the 80, 50, and 20% cases using a paired *t*-test(*df* = 22) comparing STIM vs NOSTIM cases; table 4). (C) Distribution of the agent’s performance on the held-out data over sessions for the STIM and NO STIM cases. Plotting conventions are the same as those in (B). Performance was significantly higher in the STIM condition (*p*-values were $ < 0.002$ for the 80, 50, and 20% cases using a paired *t*-test(*df* = 22) comparing STIM vs NOSTIM cases; table 5). (D) Performance of the agent on the held-out data within value difference condition for the 80%, 50%, and 20% training data case. Performance was quantified as the proportion of trials correctly predicted within each value difference condition. Error bars correspond to session-wise SEM.

**Figure 6.**
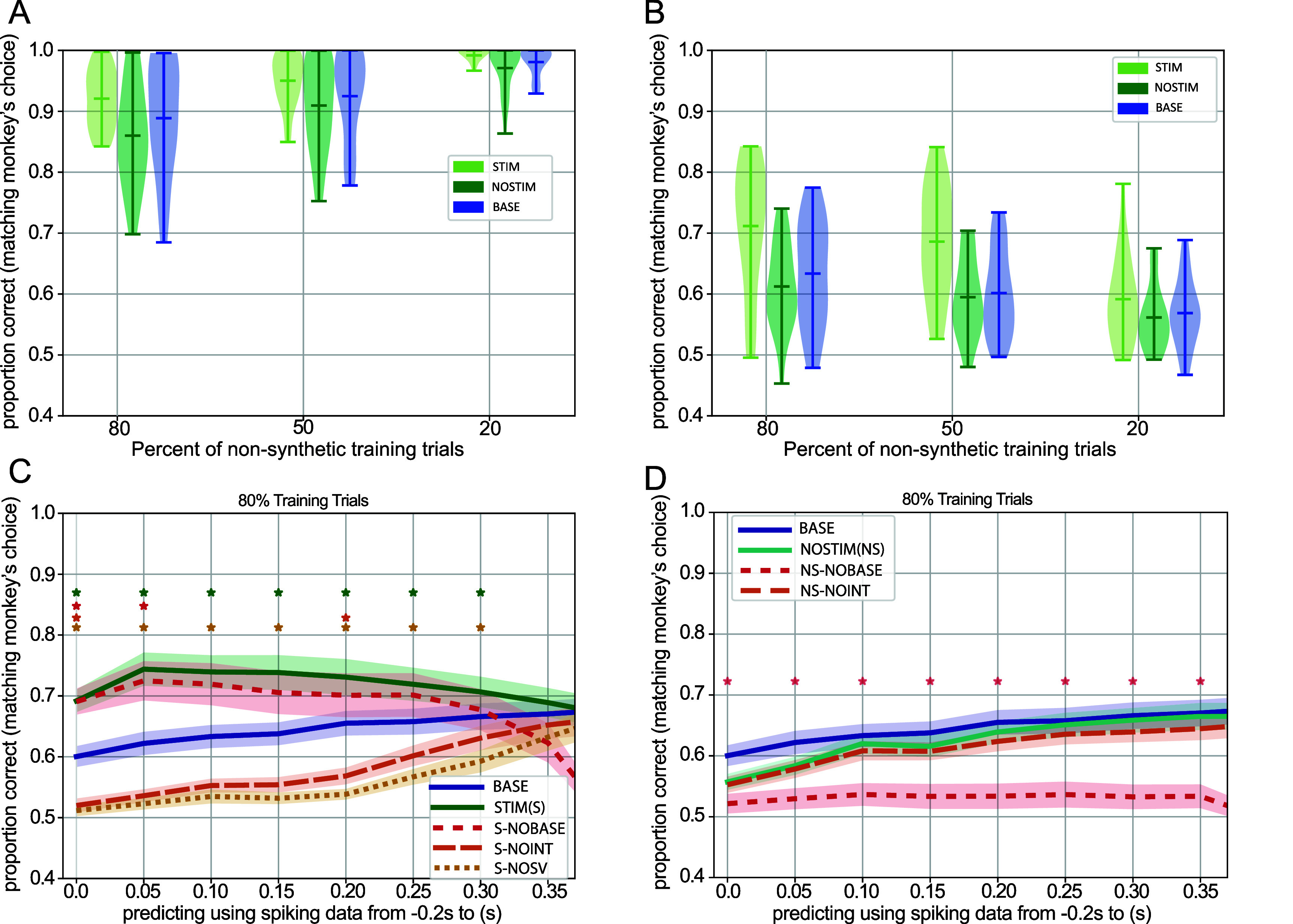
The agent with forecasting module can predict choices when only a portion of each trial’s neural data is provided. (A) Violin plots for each agent type over sessions. Performance on the training data is shown for the cases when the percentage of non-synthetic data is 80, 50, and 20% of the available trials within a dataset. All three agent types (i.e. STIM, NOSTIM, BASE) learn to make choices in an environment that varies the number of available time points the agent can access. All statistical comparisons were done using a paired *t*-test (*df* = 22). Significant differences were as follows: NOSTIM vs BASE (*p* < 0.01 for the 80% case only), NOSTIM vs STIM (*p* < 0.01 for all), and BASE vs STIM (*p* < 0.05 for all). (B) Agent performances on the held-out data for the splits shown in (A). The STIM agent performs better on the held-out data than the other model variants. All statistical comparisons were done using a Paired *t*-test (*df* = 22). Significant differences were as follows: NOSTIM vs BASE (*p* > 0.05 for all), NOSTIM vs STIM (*p* < 0.003 for all), and BASE vs STIM (*p* < 0.02 for all). (C) Average performance (correct choice prediction) over sessions in the held-out data of the agent trained using only a portion of neural data (80% training data case). The *x*-axis gives the upper limit (in seconds) of the neural data provided to the agent in each trial (the lower limit was −0.2 s for all). The model cases within the legend: BASE: BASE agent (a version of the Core model which does not have a forecasting module but is trained in the environment that varies the number of available temporal components), STIM: agent with forecasting module, with the stimulus vector provided during both training and testing, S-NOBASE: The STIM agent with the Core module contribution removed during testing, S-NOINT: the STIM agent with the forecasting module’s contribution removed during testing, S-NOSV: forecasting agent when the STIM agent with the stimulus vector not provided during testing. Stars indicate statistical significance (*p* < 0.001) when comparing each case to the BASE model performance (Statistical comparisons were done using a paired *t*-test (*df* = 22) at each time point). (D) Similar to (C). The model cases within the legend: BASE: Same as in (C), NOSTIM: agent with forecasting module, with no stimulus vector provided during either training or testing, NS-NOBASE: the NOSTIM agent with the Core module’s contribution removed during testing, NS-NOINT: The NOSTIM agent with the forecasting module’s contribution removed during testing. Stars indicate statistical significance when comparing each case to the BASE model performance (*p* < 0.001).Statistical comparisons were done using a paired *t*-test (*df* = 22) at each time point.

Environment 3 (used in figures 5 and 6)

**Environment definition** Similar to Environment 1, the agent was required to predict which of the two targets the monkey chose. However, the agent is instructed to use only a portion of the neural activity that was observed up to a time point *t* < *T* on a trial. The portion of the trial varies from trial to trial but is always a continuous blocks of time. Once the agent makes a prediction, a new trial is presented.

**State definition** A state within this environment consists of the following: neural activity (with uniform noise; see Environment 1 for details), a label indicating the first target observed, a label indicating the second target observed, and a mask indicating the neural activity up to a time point *t* < *T* that the agent can use to make its predictions. We randomly varied the parts of the time series that are masked but in all cases the masks were continuous blocks of time, consistent with the goal of designing an agent to predict choices based on the activity observed up to a given time point. We never consider providing masks to the agent in which random non-consecutive time bins were masked.

**Reward definition** The agent receives a +1 when it correctly chooses the appropriate action and 0 otherwise.

**Training details** We considered both the Core model architecture and the Internal model architecture for this environment (see Model architecture section (2.5) for more details). Both architectures were trained using the same *Q* learning approach described in Environment 1. We also used a discount factor *g* = 0 for this environment. For the Core model architecture, we trained in the same manner as Environment 1. For agents with the Internal model architecture (figure 5(A)), we used a two-step training process. The first step involved training an Internal model to forecast neural activity. The Internal model is a variational autoencoder [19] that is trained to reconstruct neural activity using only the training data (i.e. not the held-out data). At each time step *t* in the multivariate time series, the ELBO (evidence lower bound) loss was computed. We trained this autoencoder by minimizing the negative sum of the ELBO loss terms computed at each time *t*. We used an Adam optimizer for this process. After training the Internal model, the second step was to train the decision-making agent. The agent was trained as described above with the Internal model’s weights held fixed (i.e. frozen). With the Internal model held fixed, the agent uses this model to reconstruct the provided neural data and relies on permitted portions of the reconstructions to make choice predictions (see Model architecture section (2.5) for details on data masking). The training process was performed until the agent’s performance converged.

### 2.5. Model architecture

We considered two model architectures. The objective of both models was to process multivariate time series data and extract the relevant neural signals needed to predict the monkey’s choice. All modeling was done using Pytorch 2.1.1 and scikit-learn 1.2.0.

**Figure 7.**
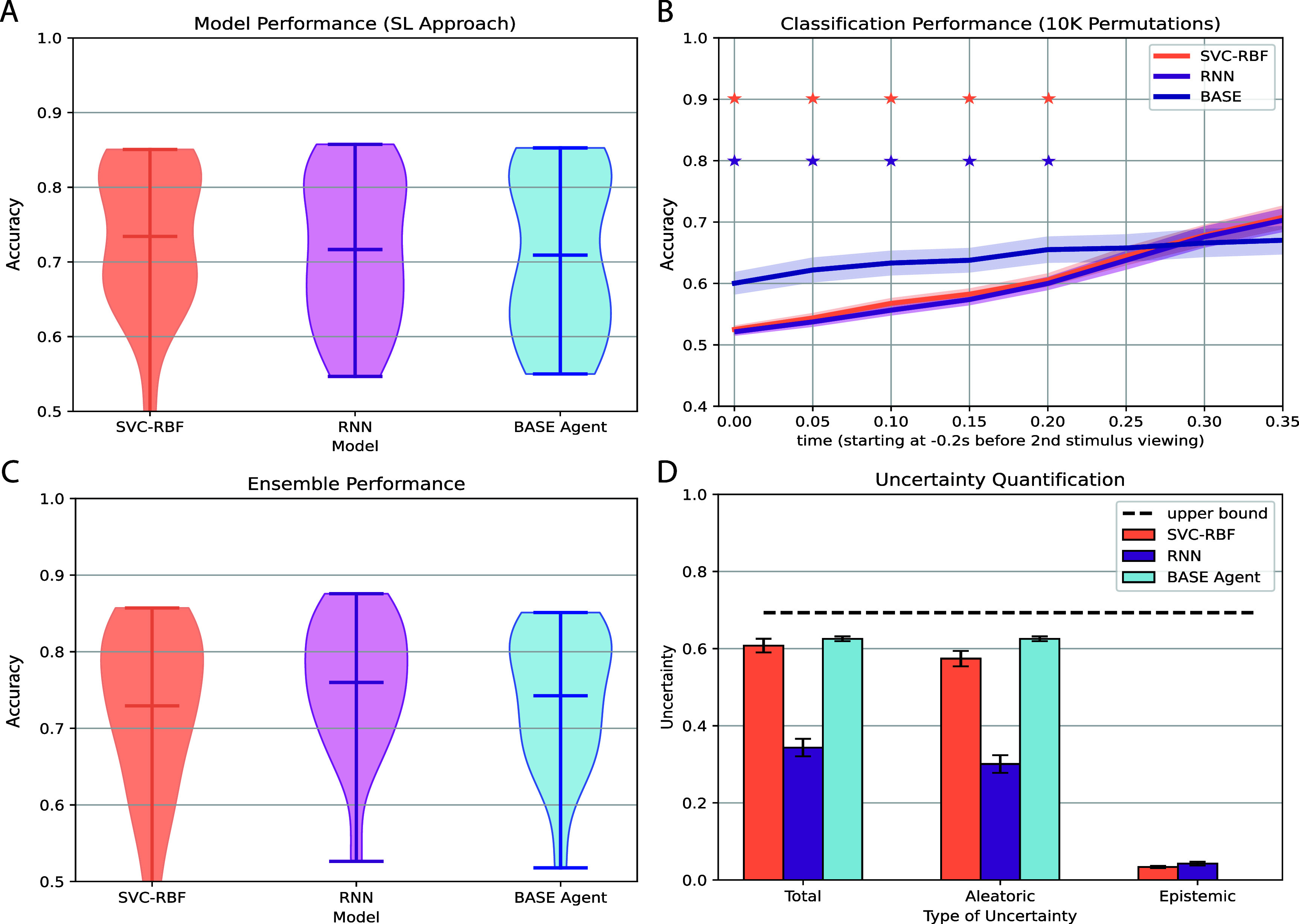
Supervised learning approaches extract similar neural activity patterns and are influenced by the same sources of uncertainty as the proposed RL approach (A) Distribution of decoding performance over sessions for a support vector machine and recurrent neural network trained using supervised learning. The distribution of decoding performance for the BASE agent is the same as shown in figure 2(D) for the 80$\%$ of the training data case. We only considered the case when 80$\%$ of the training data was used in training. There were no statistically significant differences between the decoders (*p* > 0.05 for the SVC-RBF vs RNN, SVC-RBF vs BASE agent, and RNN vs BASE agent comparisons using a paired *t*-test (*df* = 22); table 8). (B) Average decoding performance of both decoders over sessions from (A) when provided only a portion of the time series data (like figures 6(C) and (D)). We assessed choice decoding performance after permuting the parts of the time series that occurred after the upper limit (10 K permutations).Stars at each time point indicate significant difference ( *p* < 0.05) from the BASE model by paired *t*-test (*df* = 22). (C) Distribution of decoding performance on the held-out data over sessions for ensembles of each decoder type. Each ensemble consists of 5 separately trained decoders. There were no statistically significant differences between the ensembles (*p* > 0.05 by paired *t*-test (df = 22) for the SVC-RBF vs RNN, SVC-RBF vs BASE agent, and RNN vs BASE agent comparisons; table 9). (D) Average uncertainty estimates for each decoder type. Error bars correspond to SEM over sessions. The upper bound is computed as the log(*K*) where *K* is the number of classes. For this scenario, *K* = 2. There is a statistically significant difference between aleatoric and epistemic uncertainty for all model types (*p* < 0.05 for the SVC-RBF, RNN, and BASE agent models using a paired *t*-test(*df* = 22) to compare aleatoric vs epistemic uncertainty; table 10).

Core model architecture The first model architecture used a single transformer encoder module to interpret the neural data. We refer to this model as the ‘Core model/agent’ or ‘BASE agent’ in figures 5–7. The architecture for the ‘Core model’ (figure 2; figure S2 in Supplementary data section for intuitive explanation) consisted of first embedding the neural data *X* using a linear layer

\begin{align*} E = W_\mathrm{Embed}*X + b\end{align*}

and then applying positional encoding (using a series of sine and cosine functions) to maintain the temporal ordering as done in [20]. Through parameter testing, we found that an embedding dimension of 100 to yield stable performance across sessions.

Using a Bert style approach [21], the output along with a class token is then fed into a 2-layer transformer encoder. Each transformer encoder layer can be described by the following set of equations

\begin{align*} Q &amp;= W_{Q}*E + b_{Q}\end{align*}

\begin{align*} K &amp;= W_{K}*E + b_{K}\end{align*}

\begin{align*} V &amp;= W_{V}*E + b_{V}\end{align*}

\begin{align*} Y &amp;= \mathrm{Softmax}\left(\frac{QK^{T}}{\sqrt{d_{k}}}\right)V\end{align*}

\begin{align*} Z &amp;= \mathrm{LayerNorm}\left[Y\left(E\right) + E\right]\end{align*}

\begin{align*} \chi &amp;= \mathrm{LayerNorm}\left[\mathrm{MLP}\left(Z\right) + Z\right]\end{align*}

where LayerNorm refers to layer normalization. In the above equations, *Q*,*K*,*V* correspond to the query, key, and value matrices defined in the original work [20] and *d*_*k*_ is the number of rows of *K*. The first dimension (i.e. the dimension corresponding to the class token position) is then extracted and used to compute the *Q* values corresponding to the possible actions that the agent could take. The layer that outputs the predicted *Q* values is a linear layer. The action with the largest *Q* value is the one taken by the agent. The agent trained to interact with Environment 2 uses the same architecture, except that the following changes were made: (1) the Transformer encoder is updated separately for it to learn a lower-dimensional state representation (see training details in Environment 2 section), (2) the *Q* value prediction head was changed to the following:

\begin{align*} \tilde{Q} = W_{2}*f\left(W_{1}*\chi_{1} + b_{1}\right) + b_{2}\end{align*}

where $f = \mathrm{RELU}()$ is the rectified linear unit activation function and *χ*_1_ is the first dimension corresponding to the class token.

Internal model architecture We include a module that forecasts neural activity based on a combination of current activity and information about the stimulus. The second model architecture that we use is like the Core Model, but is equipped with an Internal model for neural forecasting. The Internal model provides the agent with a forecast of future neural activity based on the neural activity observed up to a time point $t < T$ and information about the trial condition. We used the Stochastic Recurrent Nueral Network (SRNN) [19] architecture for this purpose (see above). The underlying expressions that represent the forecasting module are given below. We use the notation provided in [22] to describe the original model [19]. Let $x_{t-1}$ be the neural activity and $h_{t-1}$ be the output from the hidden layer at time point *t* *− 1*. To compute the output from the hidden layer at time *t*, we provide as input both $x_{t-1}$ and $h_{t-1}$ into the recurrent layer represented by *d*_*h*_.

Deviating from the original model, we provided a stimulus vector *s*_*t*_, representing the identity of the targets available at time *t*. We concatenated this stimulus vector to the input vector *x*_*t*_ and provided this composite representation as the input to the recurrent layer *d*_*h*_,

\begin{align*} h_{t} = d_{h}\left(\mathrm{concat}\left(x_{t-1},s_{t-1}\right),h_{t-1}\right).\end{align*}

Because the forecasting module is a variational autoencoder, we leverage the output from the hidden layer to approximate the mean *µ* and standard deviation *σ* of the distribution from which we sample the latent vector *z*. We assume that the underlying conditional probability distribution *P* estimated using a model parameterized by *θ* is a multi-variate normal distribution,

\begin{align*} P_{\theta_{t}}\left(z_{t}|z_{t-1},h_{t}\right) &amp;= N(z_{t};\mu_{\theta_{z}}\left(z_{t-1},h_{t}\right),\nonumber\\ &amp;\quad \times \mathrm{diag}\left\{\sigma^{2}_{\theta_{z}}\left(z_{t-1},h_{t}\right) \right\}).\end{align*}

To compute the mean and standard deviation for this distribution at time *t*, we provide the latent vector (derived using the reparameterization trick) from time *t* − 1 and the output from the hidden layer at time *t*. Both the latent vector at time *t* − 1 and the hidden layer’s output at time *t* are provided to the function *d*_*z*_ which represents the encoder component of the autoencoder,

\begin{align*} \left[\mu_{\theta_{z}}\left(z_{t-1},h_{t}\right),\sigma_{\theta_{z}}\left(z_{t-1},h_{t}\right)\right] = d_{z}\left(z_{t-1},h_{t}\right).\end{align*}

To compute the predicted neural activity at time *t*, we provide the decoder *d*_*x*_ the latent vector *z*_*t*_ and *h*_*t*_. The decoder produces the predicted neural activity *x*_*t*_.

The amount of actual neural data provided to the model (and therefore the proportion of actual vs. forecasted data used to make predictions) was varied across trials. To train the agent over these variable temporal components, we leveraged the masking mechanism within the transformer encoder architecture to vary the time points the agent had access to on a particular trial. Each state provides the agent with a multivariate time series and two masking vectors. These vectors determine which time bins the agent can attend to in order to make its choice prediction. To encourage the agent to learn how to extract information from both modules, we designed the mask so that the forecasting module would only attend to forecasted time points. In contrast, the Core module attended to a part of the spiking data that was not forecasted. One vector is given to the transformer encoder present in the Core module. The other vector is given to the transformer encoder present in the forecasting module. For example, consider a trial in which only the first 6 of the 12 total time bins of neural data are available. In such a trial, the mask for the Core module permits the agent to attend to the first 6 time bins, while the agent’s forecasting module would predict the next 6 time bins and attend to only those six predicted bins. Once both modules have evaluated their inputs, the representations from each path are combined and used to make a choice prediction.

To provide the agent with target information, we provided the Internal model with a unique 50-dimensional fixed tag (*s*_*t*_ in equation 11) for each stimulus provided during the trial. This 50-dimensional fixed tag is implemented via assigning a vector to each stimulus, where each entry in the vector is drawn from a uniform distribution. This vector does not change throughout training. This tag allows the Internal model to condition its forecast of OFC activity on the presented stimuli. We provided the Internal model with the first stimulus vector when the portion of real neural activity occurred before the second fixation. After the second fixation, we then provided the second stimulus vector for time windows. Because information for both stimuli is available after the second fixation, we used the vector sum of the two stimulus vectors observed on a trial as the stimulus representation for both stimuli. Combining the neural activity forecast from the Internal model with the Core module architecture allowed us to consider choice predictions at earlier points in the trial. After the initial training of the Internal model, the Internal model’s weights remained fixed while the agent’s weights were allowed to update as described above.

In summary, the model (figure 5(A)) contains two processing modules that aid in choice prediction: (1) a module that uses neural activity observed up to a given time point and (2) a module that forecasts future dynamics of the trial and uses only the forecasted activity. The current and forecasted trial information are combined and used to predict the *Q* value. See figure 5(A) for a diagram of the architecture and figure S3 for an intuitive explanation in the Supplementary data section. Representations for both paths were done using a 2-layer transformer encoder module with the Internal feature dimensionality of 100 and RELU (rectified linear unit) activation for the intermediate layers. In figures 5 and 6, we consider two versions of the Internal model: (1) where the Internal model has access to the fixed tag (i.e. ‘STIM agent/model’), and (2) where the Internal model does not have access to the fixed tag (‘NOSTIM agent/model’).

### 2.6. Agent analysis

To quantify the amount of value information present in the internal representations of the agent, we decoded the associated value of the stimuli present in the trial using the agent’s internal representations. We performed this analysis using the Core model (see Model architecture section (2.5)). We extracted the 100-dimensional vector for each trial corresponding to the first dimension of the transformer encoder module output. This vector was aligned to the position of the class token. With this collection of vectors, we used linear regression to decode the value of the stimuli. Decoding was conducted using the 80% training data case introduced in figure 2. The linear regression was fit to the representations generated from the training data and tested on the representations derived from the held-out data. The violin plots in figure 4(A) show the distribution of *R*^2^ values over all neural recording sessions.

Given the difference in data preprocessing used in this work compared to the previous study [10], we verified the ability to decode both targets’ value (results in figure 4(B)). Value decoding was done with a linear regression model using the original neural data at each time bin. The decoding results reported in figure 4(B) were derived from a 5-fold CV with 10 repeats. To assess what time bins within a trial are important for the agent to predict the monkey’s choice, we performed Shapley analysis on each model [23]. This analysis was only conducted on the held-out data. Shapley analysis was done using Python’s gradient explainer function from the SHAP toolbox [23]. To assess the magnitude of each time bin’s contribution within a recording session, we computed the average of the absolute value of the estimated Shapley values.

### 2.7. Supervised learning approach

We compared the choice prediction performance of the RL-based agents described above to standard supervised-learning methods. We trained a support vector classifier (SVC) and an ordinary recurrent neural network (RNN). We examined the performance of both decoders by computing the average prediction accuracy over the test set derived from 5-Fold CV splits of the data. Data was provided to the RNN in the same format (i.e. Trials × Cells × Time) as the RL agents. To train the SVC, we collapsed the Cells × Time dimensions so that the decoder was fit to vectors of shape Cells×Time.

### 2.8. Uncertainty quantification

We performed uncertainty quantification to measure the relative contribution of data-derived and model-derived uncertainty for the RL, RNN, and SVC decoders. Uncertainty quantification was performed using the ensemble method. For each decoder type (i.e. RL agent, SVC, RNN), we trained a set of 5 decoders (i.e. our ensemble) separately on the training set to predict the choice of the monkey (i.e. a binary classification problem). Each model was then made to produce prediction probabilities for each class per trial. Predictions were made with the ensemble using the average of the five prediction scores. For the RNN and RL agents, different decoders were created via random initialization. For the SVC, different decoders were created by fitting each classifier on a non-overlapping portion of the training set. We used the following expressions to estimate total uncertainty TU, aleatoric uncertainty AU, and epistemic uncertainty EU [24, 25] where *H* corresponds to the entropy and the $p(y|x)$ corresponds to the classifier’s probability scores:

\begin{equation*} \mathrm{TU}\left(x\right) = H\left[\frac{1}{5} \sum_{i = 1}^{5}p\left(y|x\right)\right]\end{equation*}

\begin{equation*} \mathrm{AU}\left(x\right) = \frac{1}{5} \sum_{i = 1}^{5}H\left[p\left(y|x\right)\right]\end{equation*}

\begin{equation*} \mathrm{EU}\left(x\right) = \mathrm{TU}\left(x\right) - \mathrm{AU}\left(x\right).\end{equation*}

Each uncertainty type was computed for each trial within the held-out dataset. We report the average of these uncertainty types.

## 3. Results

### 3.1. Reinforcement learning approach for choice decoding

We developed an adaptive decoder to predict monkeys’ choices in a two-alternative economic decision task using the activity of concurrently recorded OFC neurons (see Reinforcement learning approach section (2.4) in the methods, Environment 1). Because different neurons were recorded in every session, it is not feasible to pre-train a model on data from one session and test it on data from another. Therefore, we split the trials randomly within a session into a training set and a test set. The fraction of trials used for training varied from 20% to 80%.

To train the neural decoder, we used a reinforcement learning approach [11]. Within an RL viewpoint, the neural decoder is an artificial agent interacting in an environment defined by neural activity. When the agent makes a prediction, the environment provides a feedback signal—a positive signal (‘1’) if the agent’s choice matches the monkey’s, and a ‘0’ otherwise—which is used to update the agent’s parameters. We trained the agent using a standard deep Q network approach [26]. Figure 2(A) shows the architecture of the agent. We first examined the agent’s performance on the training data. After a few hundred iterations, the agent learned to predict the monkey’s choices in the training set with near-perfect accuracy (figure S1A).

Although this demonstrates that the neural decoder can learn to match the choice preferences of the monkey, we sought to answer two crucial concerns related to feasibility. The first concern relates to the ability of the decoder to generalize to new trials, which is a weakness in some RL models [27]. The second concern concerns the number of trials needed to train the model to make accurate predictions, given that practical considerations limit the available training time in a BMI setting. We jointly tested these concerns by testing decoder performance in held-out trials, and by varying the number of trials in the training and held-out splits (figures 2(C) and (D)). Given the relatively low trial counts (mean 623 trials per session), we augmented the training data by creating ‘synthetic’ trials. The cell firing rates in synthetic trials are based on the original training trials but have uniform noise added to each cell’s firing rate (see methods sections on Data preprocessing (2.3) and Reinforcement learning approach (2.4)). The noise was independent for each unit. The performances on training and held-out trials, shown in figures 2(C) and (D), allow us to gauge how many non-synthetic trials are needed to train the decoder.

Performance on held-out data was lower than in the training set, but still above chance (figure 2(D)). When synthetic data were used for training, predictive accuracy degrades slightly (within 10% difference) as the percentage of training trials decreases from 80% to 20% (figure 2(D), light blue). This result suggests that we can still obtain above-chance out-of-sample performance with as few as 20% of trials (average 126 per session). In contrast, when synthetic data were not used in model training (figure 2(D), dark blue), performance in held-out data decreases significantly for the 20% training set case (figure 2(D), Synth vs NoSynth, $ p = 3.83e-4$ by *t*-test). Thus, synthetic training trials decrease the number of training trials needed to obtain good decoder performance.

We next asked how performance depended on the difference in value of the two stimuli presented, an index of trial difficulty. Shown in figure 2(E) is the proportion of trials that the model correctly predicted for each value difference, using 80%, 50%, and 20% of the non-synthetic data trials as a training set (figure 2(D), far left on the *X*-axis). We observed above-chance performance in all value differences. The above-chance performance on value difference zero trials (when neither choice is objectively correct) is notable because it implies that the agent can detect the monkeys’ subjective sense of value as it fluctuates on individual trials. Decoding performance on the high value difference trials was far less than 100% on average. This contrasts with the monkeys’ choices in such trials, which were nearly 100% in favor of the best option (e.g. [12], their figure 1). Thus, the information available to the decoder cannot wholly replicate the monkeys’ behavior even for the easiest trials. Consistent with figure 2(D), in the 20% training data case, synthetic training data increased the prediction accuracy in held-out trials (figure 2(E), right).

### 3.2. Developing decoders to perform goal-aligned actions in multi-step scenarios

Our initial example illustrated a scenario in which the choice information extracted from OFC activity was used to select the option that the monkey desired. However, the utility of these neural signals can also be realized in scenarios where the user’s neural activity does not explicitly indicate the decoder’s action, yet the actions taken by the decoder align with the user’s goal. An example of this can be found in human–robot collaboration scenarios where the robotic system learns a behavioral policy that indicates the actions that will aid the user in achieving the user’s goal. Previous work [28] demonstrated that robotic systems could be developed to use neural signals to learn a behavioral policy in a sequential collaboration scenario. Taking inspiration from this domain, we consider a collaborative version of the initial task, in which the decoder must learn to execute a sequence of actions if the monkey is to obtain the reward associated with the option he chooses (figure 3(A); see methods, Reinforcement learning approach (2.4) Environment 2). The decoder will receive the neural activity and the current sequence state information.

We considered two versions of this hypothetical scenario: (1) where a unique action sequence is mapped to each value level and (2) where the action sequence is mapped to the order in which the monkey views the target. The viewing-order mapped case allows us to consider scenarios where the agent’s actions are based solely on the choice information. A common example of this case can be found in online shopping. When a person aims to maximize their savings while choosing between two versions of an appliance, a value-based assistive system capable of executing multi-step decisions can help. This system assesses the user’s choice preferences and can perform actions such as navigating the selection screen, selecting the item on their behalf, and finding lower-priced alternatives.

Conversely, the value-mapped case allows us to explore a more nuanced scenario in which an agent’s actions are influenced by an individual’s subjective ranking of available options. An example of this case can be realized in automated health monitoring systems, where each person has unique preferences regarding activities and foods. For instance, if someone aims to maximize weight loss, a value-based assistive device can learn to identify the activities and dietary options the user prefers from the available choices. The device can then suggest and plan physical activities and dietary selections that align with the user’s weight loss goals while finding ways to enhance the overall experience, especially when the subjective ranking of the option is low.

Figures 3(B) and (C) show the decoder’s performance for both scenario types on the training and held-out data. The RL-trained decoder achieved above-chance performance in both scenario types on the training and held-out datasets. However, performance was superior in the viewing order-mapped action sequence scenario. These findings show that value-related choice information can be used in scenarios with interactive dynamics. However, the performance of such systems will vary based on how much the synergistic demands between user and agent depend on what information is encoded within the choice-related representations.

### 3.3. Interrogating the internal representations of the choice decoder

Given the decoder’s prediction capabilities on both tasks, we next asked what aspects of its input are essential to making its decision. Previously, we have shown that choice decoding from neural data could be performed at above-chance levels when using neural signals only related to the first of the two targets [10]. Therefore, we asked whether the agent makes predictions using information about one target or both. We extracted the internal representations from the trained agent from the first task (figure 1(A)) for each trial and attempted to decode the values of the two items in each trial. Because we use a transformer encoder, we use the output corresponding to the class token as the agent’s trial representation. We considered three variables in our decoding analysis: the value of the 1st target, the value of the 2nd target, and the difference between the 1st and 2nd target values. The degree to which we can decode each of these variables highlights how much target-specific information is present within the choice representation of the agent. Shown in figure 4(A) is the value decoding performance in held-out data, when using 80% of the data to train the model. We find above-chance decoding of value information from the agent’s representations for each target individually, as well as for the difference between the two targets’ values.

Next, we asked which time bins in the trial were necessary for the agent’s prediction, using a standard approach to Shapley analysis. Previous studies [10, 29, 30] have shown that value signals are dynamic and are computed time-locked to the viewing of the targets. These studies have also identified a 200 ms delay between when the monkey sees the target and when value information is first detectable in OFC (e.g. [10], figure 2). Like these prior OFC results, we could decode from the neural data the value of the first target in earlier time bins, and the second target value in later time bins (figure 4(B)), blue and orange lines). Considering these observations, we expect that later bins, i.e. after both targets have been viewed, will be more critical for the agent’s *Q* value predictions.

We examined the absolute value of the Shapley values to understand how much each feature (time bin) contributes to the *Q*-value estimates regardless of the sign. Over all sessions (figure 4(B), black curve), the agent found later time bins (${\gt}0.2$ after 2nd target viewing) to be the most informative. However, the results suggest that the agent also uses information from the early timepoints (0.1 s in figure 4(B), when only information about the first target is available) instead of exclusively relying on the last time bins (when information is available for both targets). Together, these results indicate that the RL decoder predicts choices by extracting information about the values of the targets offered in each trial.

### 3.4. Augmenting choice decoding with neural forecasting

Next, we extend the model above to consider an assistive BMI system that can anticipate a user’s abstract goals or intentions, guiding the user to act more rapidly than would otherwise be possible (see methods, Reinforcement learning approach (2.4) Environment 3). To achieve this objective, we take inspiration from human–robot interaction systems that aim to forecast the user’s actions before the robot moves [31]. Specifically, we explore a BCI system equipped with a neural forecasting module. In our hypothetical scenario, the agent is provided with a module that uses a portion of neural activity up to a given time point to forecast future neural activity and to then predict the monkey’s choice. Under the assumption that ongoing activity dynamics shape subsequent activity [32], the forecasting module would permit the system to predict the OFC’s activity before it occurs, making faster goal inferences than would be possible from native neural data alone. In the robotics scenario, this would be akin to inferring the user’s intent slightly before the user’s motor execution, giving the system more time to plan how its actions will aid the user. Thus, we constructed an agent system like the one described above, but with an Internal model whose function is to forecast future neural activity out to a maximum time *T* given the activity up to some point *t* < *T* (figure 5(A)). The maximum time *T* in this work is 400 ms after the monkey viewed the second target (i.e. the full length of the time series).

Using this forecasting module, we also consider an additional method for increasing the agent’s speed and decision accuracy by providing the Internal model with information about the targets in each trial. Providing external information about the targets allows us to consider an assistive neuro-engineering system that uses or complements one’s existing sensory capabilities to maximize the information available to (and therefore the predictions made by) the BMI agent. As described earlier, OFC’s activity depends on the visual targets presented in each trial, and the time course of OFC activity within a trial depends on when each target is viewed. Therefore, to more accurately forecast OFC activity, we provide the Internal model with information about the targets in each trial, in the form of a fixed ‘stimulus vector’ unique to each stimulus (figure 5(A), brown box). Providing the agent with the information about the targets does not trivialize the problem because a sufficient portion of trials pertain to pairs of stimuli that possess the same value. In these trials, there is no ‘objectively’ correct choice, so that choices reflect the subjective preferences of the animal [10, 12, 33]. Therefore, an important question is whether the agent’s predictive accuracy for these subjective choices is improved when stimulus information is available or improves performance only for choices with an objectively correct (higher-value) option.

Below, we assess the performance of agents using forecasting modules. First, we quantified the maximum benefit that a forecasting module could provide to the agent (i.e. choice-prediction performance upper bound; results in figure 5). We then quantified typical performance under conditions that most resemble our expected use case (results in figure 6). In both cases, we compare the performance of agents with vs. without the stimulus vector information provided to the forecasting module.

#### 3.4.1. Assessing the performance upper bound

Given neural data up to a time point ($t_{0},{\ldots}, t_{n}$), the forecasting module predicts the activity iteratively: it first predicts activity at $t_{n+1}$, and then uses the predicted data at $t_{n+1}$ to predict the activity at $t_{n+2}$, and so on until the end of the trial. Thus, any forecasting error occurring at $t_{n+1}$ propagates to subsequent time points into the entire forecasted portion of the data ($t_{n+1},{\ldots}, T$), which ultimately affects the agent’s choice decoding performance. To estimate the system’s performance upper bound, we wished to minimize the effects of these time-propagated forecasting errors.

To do so, we allowed the Internal model to provide the agent with reconstructed neural data, rather than forecasted data. Reconstructed data is obtained by giving the forecasting module the real neural data before each time point. (Because the forecasting module is an autoencoder, it is optimized to reproduce (or ‘reconstruct’) the data provided as an input. Thus, the reconstructions of neural data can be considered a quality upper bound). Using reconstructed data avoids the propagation of forecasting errors across time points and therefore minimizes the effects of the Internal model-derived errors on the agent’s choice prediction. We consider the performance when the agent uses reconstructions to make choice predictions (figure 5) as the highest performance (i.e. upper bound) achievable by this model.

We assessed the performance upper bound when the Internal model has access to both neural activity and a vector representing stimulus information (STIM agent), as well as an Internal model provided only with neural activity (NOSTIM agent). Both used synthetic data trials during training (i.e. as in the ‘Synth’ models in figure 2). Across all the recording sessions, both STIM and NOSTIM agents achieved comparable performances on the training data (figure 5(C). We next evaluated the generalizability of each agent by examining their predictions on held-out data (figure 5(D)). In both model cases, both agent types saw a considerable performance drop as the fraction of training trials decreased. Across all data partitions, the STIM agent outperformed the NOSTIM agent (figures 5(C) and (D)), and predictive performances were higher when the value difference was larger (figure 5(E)). Thus, when neural forecasts are augmented by information about the target stimuli, the agent can more accurately predict the monkeys’ choices.

#### 3.4.2. Forecasting neural data improves predictive accuracy early in a decision trial

The results in figure 5 show the impact of the neural forecasting module under ideal conditions, specifically, when we eliminate time-propagated inaccuracies that originate from forecasting neural data. In contrast, figure 6 shows the impact of the forecasting module under more realistic conditions, specifically, when only an initial segment of neural data is provided, such that forecasting errors at each time step propagate into future time steps. To test this, we trained the agent using trials in which only an initial portion of real neural data is provided to the system (e.g. the first 6 out of 12 total time bins, variable across trials), requiring the Internal model to forecast the remaining data. We trained agents with and without stimulus information on every trial (STIM and NOSTIM, respectively). In addition, to assess the impact of the forecasting module, we trained an agent with no forecasting module (like the Core model in figure 2) to make choices in the same environment as the STIM and NOSTIM agents (i.e. with a variable initial segment of neural data provided). We refer to this agent as the BASE agent.

In the training data, the STIM agent had the highest performance on average for all non-synthetic data splits (figure 6(A)). Despite not having access to a forecasting module, the BASE agent slightly outperformed the NOSTIM agent (see table 6 in the Supplementary data section for statistics), suggesting that forecasted activity lacking task-relevant information may negatively impact the training process. In the held-out data, the STIM agent also outperformed the other agent types, with no significant difference in performance between the NOSTIM agent and the BASE agent (figure 6(B), table 7). These results suggest that the forecasting module provided no benefit to choice prediction without information about the task stimuli (i.e. the stimulus vector).

We next sought to characterize the impact of the forecasting module by assessing the agent’s performance as a function of how much initial neural data was provided (figure 6(C). In addition, we evaluated the contribution of individual model components by selectively ‘ablating’ architectural components during testing (figure 6(D)). We focused our analysis on the 80% training data case. The predictive performance of the BASE agent gradually increases as more data is provided (figure 6(C), blue line), consistent with the observation that later time bins are more informative (figure 4(B)). In contrast, the STIM agent’s performance is high regardless of how much initial data is provided (figure 6(C), green line), indicating how informative the Internal model’s forecasted activity is for predicting choice even at early time points in the trial.

As we removed modules from the STIM agent, we observed complementary effects on performance. Removing the Core module contribution had little impact in early portions of the time window, but its absence decreased performance at later time points in the trial (figure 6(C), red line). A complementary pattern was observed after removing either the forecasting module or the stimulus vector: performance was near chance at early portions of the time window and improved at later time points (figure 6(C), gold and brown lines). These results demonstrate that the STIM agent depends mainly on the forecasting module at early time points and uses a combination of actual neural signals (from the Core module) and forecasted neural signals as time elapses in the trial.

The NOSTIM agent, trained with a forecasting module but no stimulus vector, had performance that was almost entirely dependent on the Core module (figure 6(D)). Removing the ability to forecast had virtually no effect on performance (figure 6(D), red line vs green line). However, when the Core module was removed, the NOSTIM agent’s performance dropped to chance levels. These findings suggest that forecasting OFC activity does not help predict behavior unless task information is available to the agent. However, when additional task information is available, predicting behavior earlier within the trial is possible.

### 3.5. Comparing a supervised learning approach to the proposed RL construction

Thus far, we have demonstrated that an RL-trained neural decoder can predict the monkeys’ choices in single-action decisions (i.e. Environments 1 $\&amp;$ 3). However, we recognize that a more straightforward approach to developing neural decoders is through the use of supervised learning (SL). Previous work [9, 10] has demonstrated that simple linear decoders trained using an SL approach are sufficient at decoding value and choice.

However, our problem description differs from theirs in that (1) we train a model to make choice predictions using multivariate time series data and (2) we are accounting for decoders that can handle multi-step scenarios. To shed light on how model considerations and training approaches lead to differences in prediction performance, we trained a SVC and an ordinary RNN using a standard SL training process (i.e. no data augmentation and no varying trial lengths) to predict choices given multivariate time series data. Both models have been shown to perform well in neural decoding contexts [34].

Figure 7(A) shows the decoding performance on held-out data for the SVC and RNN compared to our Environment 1-trained BASE agent (figure 2). Both the SVC and RNN had slightly higher performance on average, but these differences were not statistically significant. (*p* > 0.05; table 8). Thus, when the full neural time series is available, our RL construction produces decoders whose performance is statistically indistinguishable from those trained using typical supervised learning methods.

In contrast, we find the RL-based decoder has higher accuracy than SL methods when neural data are only available up to a time point *t* < *T*, forcing the agent to choose with limited information. Using the evaluation criteria from figures 6(C) and (D), we compute the accuracy of the predictions on the test set as we provide each model a time series up to a specific time point *T*. Figure 7(B) shows that both SL models (i.e. SVC and RNN) had near chance performance when forced to predict the choice when the monkey views the second target and increases as more of the time series is provided. This result differs from the performance of the Environment 3-trained BASE agent, which showed above-chance prediction performance early on (7B blue curve). Prior work [10] has shown that both early and late-stage choice information is reflected in OFC neural activity. Thus, we show that an RL-based decoder can make accurate decisions faster than SL-based methods when given only an early segment of neural data, whereas RL and SL accuracy are equivalent when all neural data are available.

### 3.6. Measuring sources of uncertainty within the neural choice decoders

As a follow-up to the comparison between decoders (figures 7(A) and (B)), we asked whether the decoding methods differed in terms of the sources of decoder uncertainty that limit predictive accuracy [24, 25, 35]. Particularly, we want to quantify how much of the uncertainty is attributable to the model’s inability to capture the relationship between the neural activity and the choice (i.e. epistemic uncertainty) or to variability within the data (i.e. aleatoric uncertainty). This approach allows us to assess whether the lack of unique performance differences results from poor model selection (i.e. incorrect assumptions about the relationship between neural activity and choice) or irreducible data properties that will affect all models regardless of training approach.

A common approach to estimating both types of uncertainty is to create an ensemble of classifiers and to compute the entropy among the classifiers’ predictive distributions [24, 25, 35]. Shown in figure 7(C) is the prediction performance in Environment 1 of an ensemble consisting of each model type. Again, we observe similar performances across all 3 model types despite differences in model construction/architecture and training approach (figure 7(C); *p* > 0.05 table 9). We also observed that across all models, the aleatoric uncertainty was higher than the epistemic uncertainty (figure 7(D); *p* < 0.05 table 10). This suggests that the uncertainty in each decoder’s predictions predominantly stems from the variability within the data. Such high uncertainty negatively impacts each model’s prediction performance.

## 4. Discussion

The purpose of neuro-engineering systems is to aid users in accomplishing their goals. Here, we explored the use of economic value information in a hypothetical neuro-engineering system designed to interface with the user at the level of underlying motivations rather than actions. Using computational approaches with offline data, we demonstrate that constructing a value-based system using RL approaches is feasible. Within a task in which a monkey is provided two options to choose from, we constructed an agent that could be trained to extract value-related information from the activity of neural ensembles in OFC and to then predict the monkey’s choice above chance, even in trials where there was no objectively superior option. In a hypothetical collaboration-based task, we showed that the decoder could learn to predict action sequences that align with the monkeys’ choices. We also demonstrated that such a system can be trained with neural and performance data from only a small number of trials (mean 126 per session), and that the system can be augmented to make faster predictions with the addition of a neural data forecasting module with access to sensory-related signals (e.g. from a hypothetical sensory assistive device) as part of a multi-modal or multi-source BMI architecture.

Not only did we observe the ability to make faster predictions when information about sensory stimuli is provided (figure 6(C)), but our analysis also revealed how important this information is to forecast neural activity. Forecasting neural data without providing external sensory-related signals offered negligible contributions to choice predictions early in a trial (figure 6(D)). Given the optimization procedure used (i.e. RL), the ablation analyses (figures 6(C) and (D)) showed that the agents trained with stimulus information learned to predict choice using information from the forecasting module rather than the Core module (i.e. the actual neural activity). These findings suggest that forecasting OFC activity offers little behaviorally predictive information unless informed by additional information (e.g. sensory variables).

### 4.1. Limitations in our approach

Although our results argue for the feasibility of value-based BMI applications, our approach has limitations that leave some important questions unresolved. One such question is the decoder’s stability over time. Because the original trial order was not preserved, we could not test whether decoders’ predictive performance changed over the course of a session. Not preserving trial ordering also prevents us from thoroughly assessing how our preprocessing approach (determined by the training trials’ statistical properties) would influence decoder stability.

A second important question is whether the decoder would perform equally well when provided with a significantly longer segment of neural activity. In the current study, we used a relatively brief temporal window (e.g. from second target viewing) known to contain value- and choice-relevant neural signals. A real-world BMI system would need to sample neural activity over a longer temporal window that may contain a mixture of both relevant and irrelevant signals. Given our decision to use a transformer architecture, we surmise that the decoder should be able to learn to identify and act upon the choice-related activity patterns and to ignore irrelevant activity.

The previous two limitations highlight issues relevant to any BMI system. However, a problem unique to value-based systems is the need to train on neural activity related to comparing two items. In the current behavioral paradigm, it was possible to train the decoder on all possible item comparisons (12 unique stimuli, 132 unique combinations of first- and second-viewed items). However, this is a relatively large number of unique conditions compared to those used to train motor-based BMIs. Moreover, in real-world settings, it is not feasible to train over all possible comparisons. In future studies, we will explore decoders designed to predict choices on trials containing a stimulus familiar to the monkey but unobserved by the decoder during training, or on trials with stimuli novel to both the decoder and the monkey.

In the decoder trained to execute goal-aligned action sequences, we observed good performance for choice-mapped sequences, but relatively low performance for the value-mapped case (figure 3(C)). Based on the state representation learning objective in the training process, we conclude that the limiting factor for the choice-mapped case is the ability to extract the choice preferences from the neural activity. Conversely, the limiting factor for the value-mapped case appears to be the ability to extract value information from the choice-optimized representations. The inability to accurately extract the target value from the choice-optimized representations significantly restricts a decoder’s capacity to adjust its actions according to the user’s subjective preferences. One practical solution may be to incorporate multiple auxiliary tasks into the training approach, each aligned to the different unique features of an item that inform a user’s preferences (e.g. the specific size, shape, or texture of a goal item). Another solution is to consider training approaches that factor in uncertainty estimation [35]. In this work, we performed uncertainty estimation post hoc, independent of decoder training and testing (figures 7(C) and (D)). In future work, we will consider training methods that encourage the decoder to act conservatively in uncertain scenarios, thus prioritizing user safety over potentially misguided actions.

### 4.2. Practical considerations for improving model performance

Within this study, we explored the capabilities of a decoder with access to noiseless information about the task stimuli (for motivation, see the section ‘Leveraging multi-source signals in BMI’ (4.4) below). Despite providing a noiseless stimulus signal, the system’s predictive performance was well below 100%, even for trials with large value differences between stimuli. Thus, our results could be viewed as a performance upper bound for making early-in-trial behavioral predictions using neural forecasting in this data set. Considering these results, several practical strategies can be implemented to improve decoding performance.

Considering the reported properties of neural populations in the OFC (e.g. low-noise correlations [36]), we expect that performance would be improved by recording from a larger sample of cells—i.e. by providing more independent estimates of the subjective representation of item value. In addition, previous studies have shown that supplementing neuron-level signals with concurrently measured local field potentials can increase value-decoding accuracy [9, 33]. Another step that can indirectly improve decoding performance is using chronic implants. Chronic recordings offer relatively stable access to the same cells from session to session, facilitating pre-training of the decoders using previously collected data. Finally, one could consider recording from a different brain area that also represents value-related information(e.g. amygdala [37], posterior parietal cortex [38]).

### 4.3. An argument for the RL approach

Within our initial example (figure 1), we considered a simple form of goal-directed behavior that can be accomplished in a single stage by assigning values to two stimuli. We showed that in this relatively constrained behavioral paradigm, SL-trained decoders produced performance values indistinguishable from RL-trained decoders (figure 7(A)). However, we also demonstrated that an SL-trained decoder struggles to make accurate choice predictions with access only to early-stage choice information compared to our RL-trained decoder (figure 7(B)). This performance observation reflects the SL models’ parameters being overly weighted towards the late-stage choice information, which is the most predictive of the monkeys’ choices.

Despite this limitation, an RL decoding approach is ideal for augmenting goal-directed behavior because it is capable of the flexible inferences that underlie complex goal-based decisions in real-world settings. In real-world settings, goals must be pursued in more complex hierarchical situations that can have multiple steps and that can require making inferences using partial information [39]. In addition, goals can change according to context or internal demands. Thus, a successful BMI for goal-directed behavior must be flexible. It is for these reasons, along with the adaptability that the framework affords us, that we used an RL decoding approach. Within our second hypothetical scenario (figure 3), we presented a multi-step situation, where the agent had to determine the monkey’s choice and then decide what actions to take to allow the monkey to obtain the reward. This example demonstrated how a reinforcement learning approach can aid in developing decoders that make and utilize choice predictions in task scenarios with intermediate steps. Future work will focus on using tasks with a hierarchical organization like those discussed in [40] and exploring the assistive capabilities of RL-based neuro-engineering systems.

### 4.4. Leveraging multi-source signals in BMI

We explored an agent that forecasts neural dynamics (Internal model), including a variant that forecasts OFC activity given a noiseless external signal identifying the relevant stimuli. The motivation for this exercise is a hypothetical case in which the user is also assisted by a device that samples the external environment to refine the agent’s computations. Here, we implement this assistance by providing the Internal model a fixed vector uniquely defined for each stimulus, assuming that the system had immediate access to noiseless information about a stimulus when the monkey viewed the item. However, our model formulation allows for many other implementations, such as obtaining stimulus information from a brain region upstream from the value computation process rather than from an external source. In our behavioral paradigm, this could entail retrieving stimulus information from a visual region like the inferior temporal cortex (IT). Stimulus information in IT is typically evident at shorter latencies than value information in OFC [41], meaning that these signals could render faster decisions than those possible when relying on OFC alone.

Despite our approach’s limitations, the results from the agent with a forecasting module (figures 5 and 6) show the potential utility of OFC-decoded signals being paired with other regions. We will briefly outline two potential use cases in this vein. One is pairing OFC value signals with motor or pre-motor cortex signals to augment motor plans. For example, the finding that decision reaction times are faster when selecting high-value options [42] could be incorporated into motor BMI to adjust the speed of a BMI-driven effector such as a robotic limb. Another potential use case is pairing value signals with regions that reflect other decision-related or cognitive signals. In line with previous human-robot interaction studies [43], our sequence learning example (figure 3) could be extended to simultaneously monitor neural value and error-related signals to incorporate internally derived feedback on intermediate actions.

## Data Availability

Data will be shared at a future date pending appropriate cleaning, curation, and documentation. Links to the data and code will be available on the lab github (https://github.com/McGintyLab) and the lab website, (https://www.mcgintylab.org/). Prior to public sharing, data will be made available upon reasonable request to the corresponding author. No new data were created or analysed in this study.

## References

[1] Velliste M, Perel S, Spalding M C, Whitford A S, Schwartz A B (2008). Cortical control of a prosthetic arm for self-feeding. Nature.

[2] Irwin Z (2017). Neural control of finger movement via intracortical brain–machine interface. J. Neural Eng..

[3] Gao S, Wang Y, Gao X, Hong B (2014). Visual and auditory brain–computer interfaces. IEEE Trans. Biomed. Eng..

[4] Johnston R, Abbass M, Corrigan B, Gulli R, Martinez-Trujillo J, Sachs A (2023). Decoding spatial locations from primate lateral prefrontal cortex neural activity during virtual navigation. J. Neural Eng..

[5] Card N S (2024). An accurate and rapidly calibrating speech neuroprosthesis. N. Engl. J. Med..

[6] Castegnetti G, Zurita M, De Martino B (2021). How usefulness shapes neural representations during goal-directed behavior. Sci. Adv..

[7] Padoa-Schioppa C, Assad J A (2006). Neurons in the orbitofrontal cortex encode economic value. Nature.

[8] Rudebeck P H, Murray E A (2011). Dissociable effects of subtotal lesions within the macaque orbital prefrontal cortex on reward-guided behavior. J. Neurosci..

[9] Rich E L, Wallis J D (2016). Decoding subjective decisions from orbitofrontal cortex. Nat. Neurosci..

[10] McGinty V B, Lupkin S M (2023). Behavioral read-out from population value signals in primate orbitofrontal cortex. Nat. Neurosci..

[11] Pohlmeyer E A, Mahmoudi B, Geng S, Prins N W, Sanchez J C (2014). Using reinforcement learning to provide stable brain-machine interface control despite neural input reorganization. PLoS One.

[12] Lupkin S M, McGinty V B (2023). Monkeys exhibit human-like gaze biases in economic decisions. eLife.

[13] Holmstrom L, Koistinen P (1992). Using additive noise in back-propagation training. IEEE Trans. Neural Netw..

[14] Kingma D P, Ba J (2014). Adam: a method for stochastic optimization. https://arxiv.org/abs/14126980.

[15] Van Hasselt H, Guez A, Silver D (2016). Deep reinforcement learning with double Q-learning.

[16] Raffin A, Hill A, Traoré R, Lesort T, Díaz-Rodríguez N, Filliat D (2019). Decoupling feature extraction from policy learning: assessing benefits of state representation learning in goal based robotics. https://arxiv.org/abs/190108651.

[17] Horita L R, Nakamura A T, Wolf D F, Junior V G (2021). Improving multi-goal and target-driven reinforcement learning with supervised auxiliary task.

[18] Kumar A, Zhou A, Tucker G, Levine S (2020). Conservative Q-learning for offline reinforcement learning.

[19] Fraccaro M, Sønderby S K, Paquet U, Winther O (2016). Sequential neural models with Stochastic layers.

[20] Vaswani A, Shazeer N, Parmar N, Uszkoreit J, Jones L, Gomez A N (2017). Attention is all you need.

[21] Devlin J B (2018). Pre-training of deep bidirectional transformers for language understanding. https://arxiv.org/abs/181004805.

[22] Girin L, Leglaive S, Bie X, Diard J, Hueber T, Alameda-Pineda X (2020). Dynamical variational autoencoders: a comprehensive review. https://arxiv.org/abs/200812595.

[23] Scott M, Su-In L (2017). A unified approach to interpreting model predictions.

[24] Thuy A, Benoit D F (2024). Fast and reliable uncertainty quantification with neural network ensembles for industrial image classification. Ann. Oper. Res..

[25] Lakshminarayanan B, Pritzel A, Blundell C (2017). Simple and scalable predictive uncertainty estimation using deep ensembles.

[26] Barto A G (2021). Reinforcement learning: an introduction by Richard’s sutton. SIAM Rev..

[27] Ghosh D, Rahme J, Kumar A, Zhang A, Adams R P, Levine S (2021). Why generalization in RL is difficult: epistemic pomdps and implicit partial observability.

[28] Ehrlich S K, Dean-Leon E, Tacca N, Armleder S, Dimova-Edeleva V, Cheng G (2023). Human-robot collaborative task planning using anticipatory brain responses. PLos One.

[29] McGinty V B, Rangel A, Newsome W T (2016). Orbitofrontal cortex value signals depend on fixation location during free viewing. Neuron.

[30] Hunt L T, Malalasekera W M N, de Berker A O, Miranda B, Farmer S F, Behrens T E J, Kennerley S W (2018). Triple dissociation of attention and decision computations across prefrontal cortex. Nat. Neurosci..

[31] Wang Z, Mülling K, Deisenroth M P, Ben Amor H, Vogt D, Schölkopf B, Peters J (2013). Probabilistic movement modeling for intention inference in human–robot interaction. Int. J. Robot. Res.

[32] Remington E D, Narain D, Hosseini E A, Jazayeri M (2018). Flexible sensorimotor computations through rapid reconfiguration of cortical dynamics. Neuron.

[33] Sharma D, Lupkin S M, McGinty V B (2025). Orbitofrontal high-gamma reflects spike-dissociable value and decision mechanisms. J. Neurosci..

[34] Glaser J I, Benjamin A S, Chowdhury R H, Perich M G, Miller L E, Kording K P (2020). Machine learning for neural decoding. Eneuro.

[35] Lockwood O, Si M (2022). A review of uncertainty for deep reinforcement learning.

[36] Conen K E, Padoa-Schioppa C (2015). Neuronal variability in orbitofrontal cortex during economic decisions. J. Neurophysiol..

[37] Stoll F M, Rudebeck P H (2024). Preferences reveal dissociable encoding across prefrontal-limbic circuits. Neuron.

[38] Musallam S, Corneil B, Greger B, Scherberger H, Andersen R A (2004). Cognitive control signals for neural prosthetics. Science.

[39] Ribas-Fernandes J J F, Shahnazian D, Holroyd C B, Botvinick M M (2019). Subgoal-and goal-related reward prediction errors in medial prefrontal cortex. J. Cogn. Neurosci..

[40] Schuck N W, Cai M B, Wilson R C, Niv Y (2016). Human orbitofrontal cortex represents a cognitive map of state space. Neuron.

[41] Kar K, Schmidt K M, DiCarlo J J (2018). Linking image-by-image population dynamics in the macaque inferior temporal cortex to core object recognition behavior. Cogn. Comput. Neurosci..

[42] Balewski Z Z, Knudsen E B, Wallis J D (2022). Fast and slow contributions to decision-making in corticostriatal circuits. Neuron.

[43] Staffa M, D’Errico L (2023). EEG-based machine learning models for emotion recognition in HRI.

